# Meigs’ Versus Pseudo-Meigs’ Syndrome: A Case of Pleural Effusion, Ascites, and Ovarian Mass

**DOI:** 10.7759/cureus.9704

**Published:** 2020-08-12

**Authors:** Paul Nguyen, Omid Yazdanpanah, Brianna Schumaker

**Affiliations:** 1 Department of Internal Medicine, Wayne State University School of Medicine, Detroit, USA; 2 Department of Obstetrics and Gynecology, Wayne State University School of Medicine, Detroit, USA

**Keywords:** meigs’ syndrome, pseudo-meigs’, pleural effusion, ascites, ovarian mass, ca-125

## Abstract

Meigs’ syndrome is classically characterized as the triad of ascites, pleural effusion, and ovarian fibroma. The incidence is not easily determined but has been described in medical literature. We report a case of a patient who presented for shortness of breath and was found to have pleural effusion, ascites, and an ovarian mass. Investigative measures were consistent with Meigs’ syndrome; however, definitive diagnosis was not able to be determined as our patient opted for symptomatic treatment rather than pursue surgical options. We discuss the pathophysiology of pleural effusion, ascites, and management of Meigs’ and pseudo-Meigs’ syndrome. Ultimately, we discuss palliative options for patients who are not ideal candidates for surgery.

## Introduction

Meigs’ syndrome is classically defined as the triad of ascites, pleural effusion, and benign ovarian fibroma. A key feature found in patients with Meigs’ syndrome is the resolution of symptoms after tumor resection [1]. Meigs’ syndrome is a rare condition that can only be diagnosed after ovarian carcinoma is ruled out. This remains a challenge as cancer antigen-125 (CA-125) is often elevated and workup findings typically mirror those of metastatic disease [2]. Meigs’ syndrome stands in contrast to pseudo-Meigs, which presents with ascites and pleural effusion in association with benign tumors of the ovary (other than fibromas) and malignant tumors. Benign tumors in this category include mucinous cystadenomas, teratomas, struma ovarii, and leiomyomas [3]. Although it is difficult to determine the incidence of Meigs’ syndrome, it has been reported that 0.20 per 100,000 women are diagnosed with ovarian sex cord stromal tumors (SCST) [4]. Of these SCSTs, fibromas account for 4% [5].

## Case presentation

**Figure 1:**
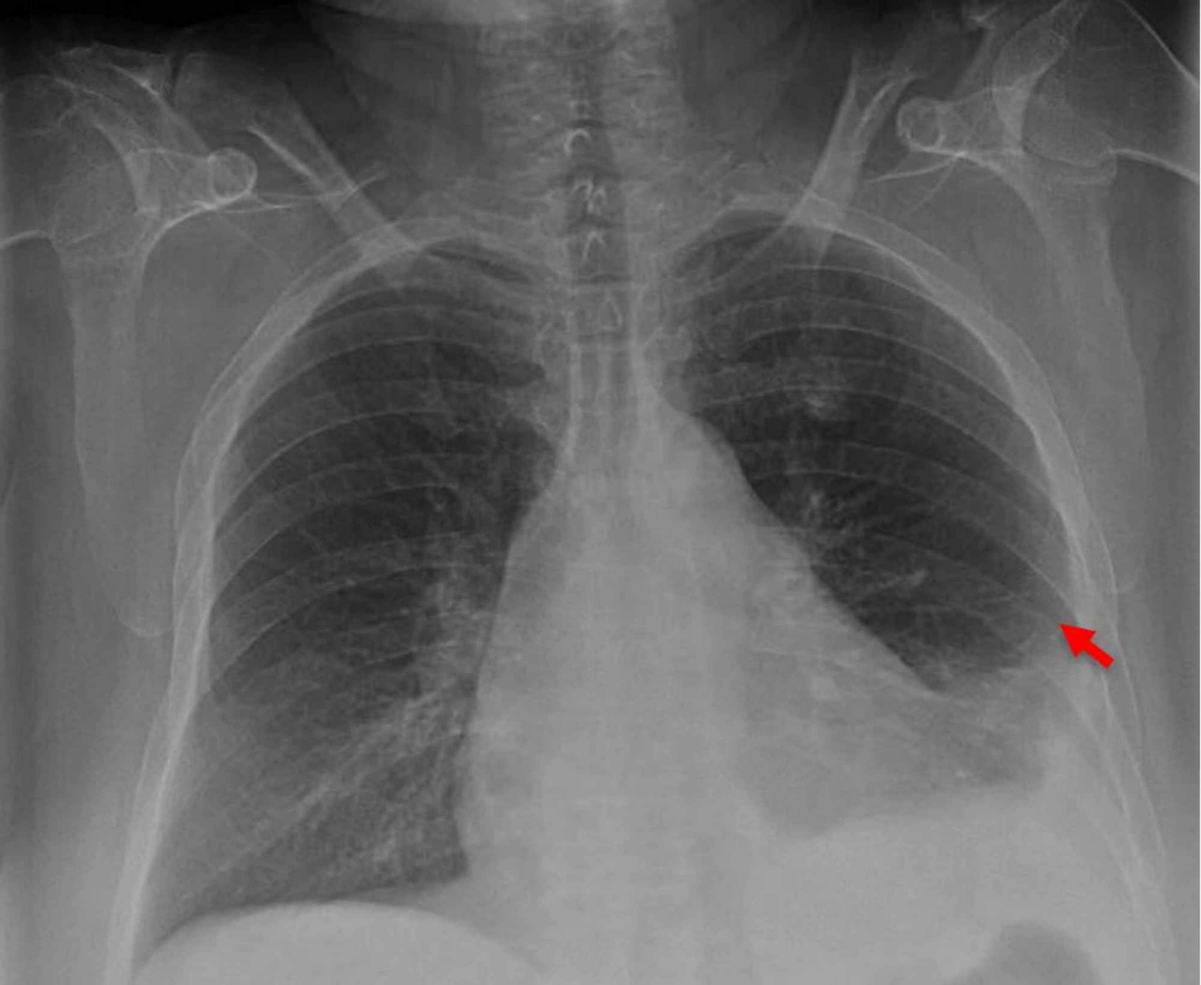
Chest x-ray showing development of left pleural effusion (red arrow).

A 58-year-old female patient with a past medical history of severe obstructive sleep apnea, chronic obstructive pulmonary disease (COPD), hypertension, and bipolar disorder presented to the emergency department (ED) for shortness of breath of two-day duration. She endorsed having a clear productive cough with fever and chills but denied any sick contacts. In the ED, the patient was tachycardic with a heart rate of 132 beats per minute, saturating 96% on room air, and hyponatremic at 127 mmol/L with a leukocytosis of 17.4 K/cumm. Chest x-ray (CXR) revealed a small left pleural effusion (Figure 1). The patient was admitted for further management of her shortness of breath and started on treatment for presumed COPD exacerbation/community acquired pneumonia.

**Figure 2:**
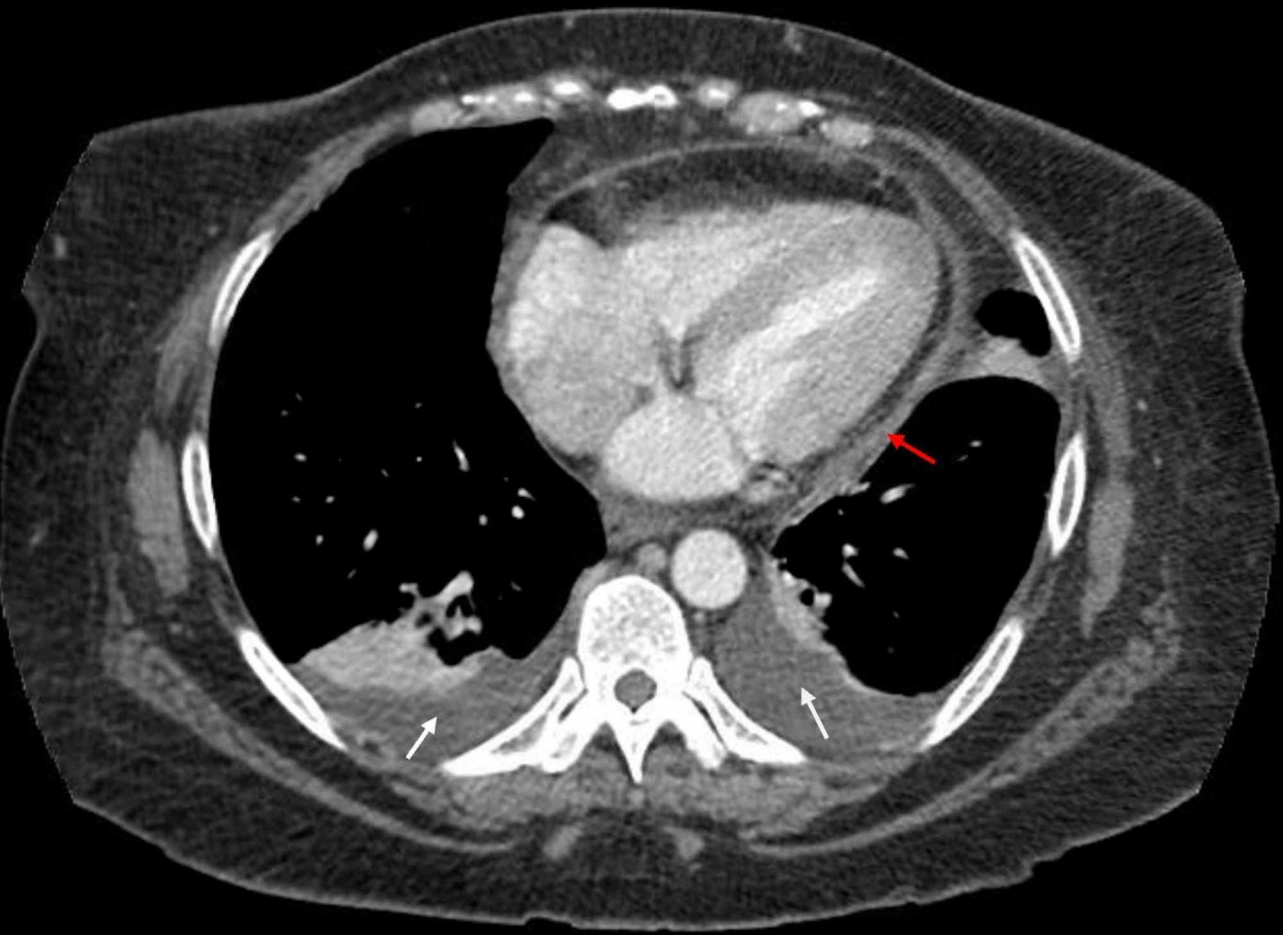
CT thorax showing pericardial effusion (red arrow) and bilateral pleural effusion (white arrow).

**Figure 3:**
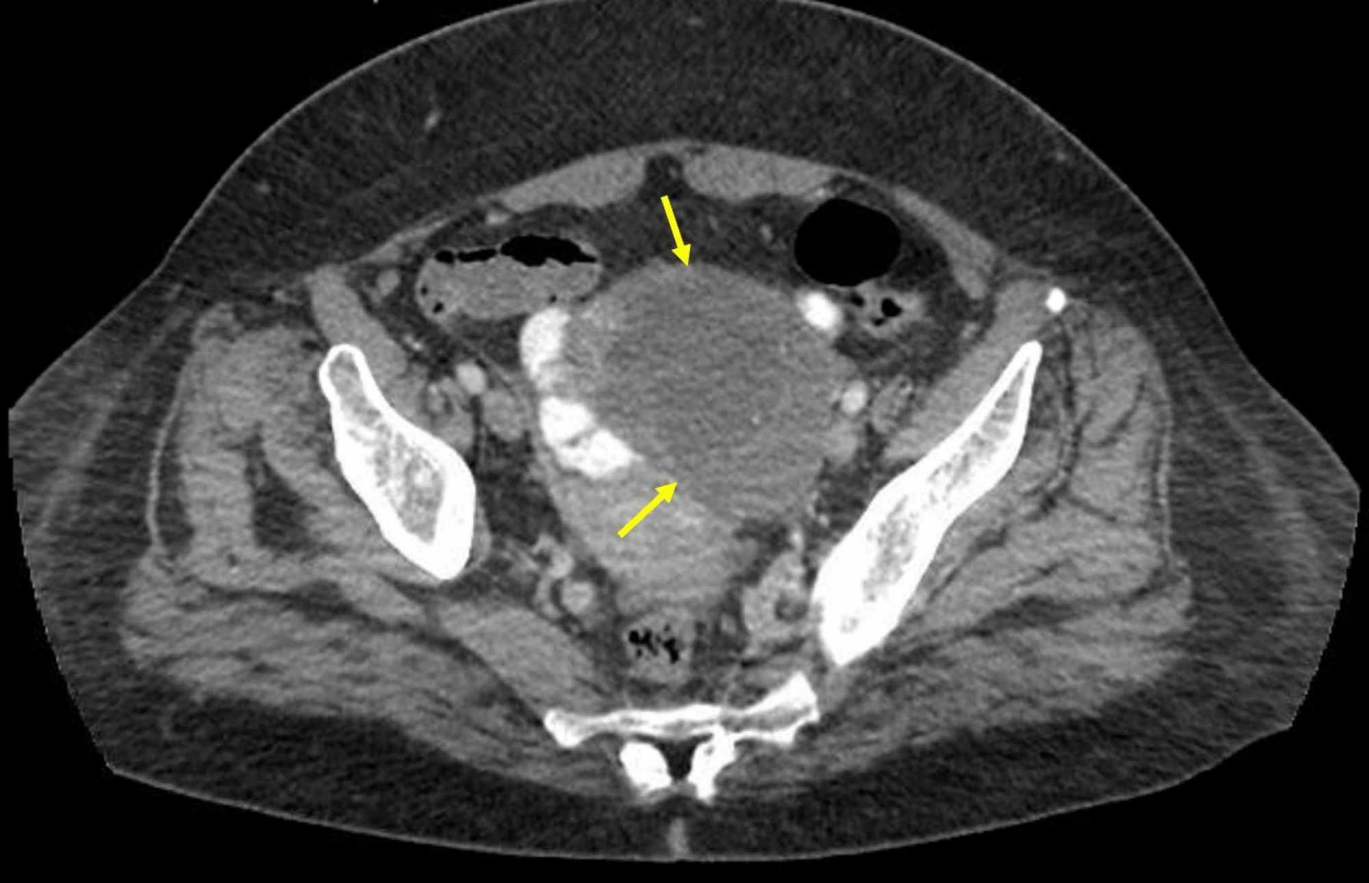
Pelvic CT showing semi solid/semi cystic left ovarian mass measuring 8.3 cm (yellow arrows).

The following day, the patient became hypoxic, saturating 86% on room air. Supplemental oxygen of two-liter nasal cannula was initiated and a repeat CXR showed bilateral pleural effusion, left greater than right. A diagnostic thoracentesis was performed, and 550 mL of cloudy yellow fluid was removed and sent for analysis. Fluid analysis revealed 1,745 nucleated cells, neutrophilic predominance of 61%, lactate dehydrogenase (LDH) 88 units/liter, protein 3.8 gm/dL, and glucose 100 mg/dL, with serum LDH 139 units/liter and protein 6.4 gm/dL. This met Light’s criteria for exudative effusion as fluid LDH to serum ratio was 0.633 and protein ratio was 0.59. Fluid cytology result was negative for malignant cells. A CT of the thorax and abdomen/pelvis was completed and showed pulmonary emphysema, bilateral pleural effusions, small pericardial effusion, nodular liver consistent with cirrhosis with small amounts of ascites and a semi-solid/semi-cystic left ovarian mass measuring 8.3 cm (Figures 2, 3).

**Figure 4:**
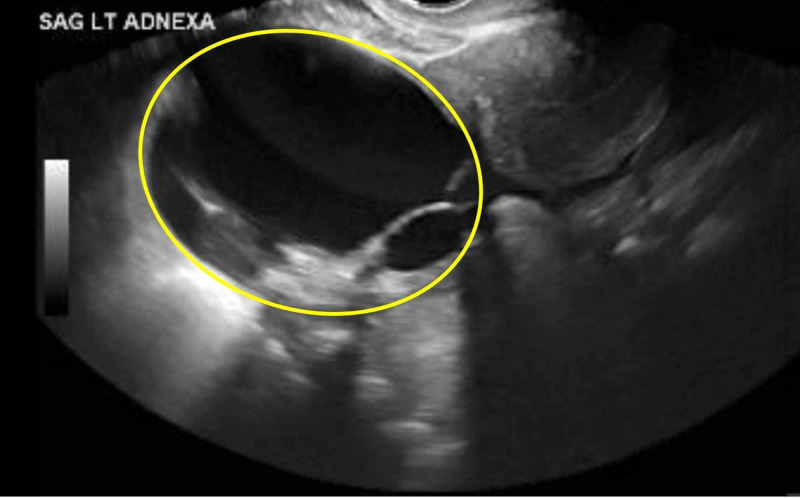
Transvaginal ultrasound showing a stable 8.6-cm multilocular left adnexal cyst with solid component (yellow circle). Color score = 1. O-RADS score = 4. Intermediate risk of malignancy (10 to <50%). O-RADS, Ovarian-Adnexal Imaging-Reporting and Data System

A transvaginal ultrasound showed a stable 8.6-cm multilocular cystic left adnexal mass (Figure 4), which was compatible with benign cystic ovarian neoplasm such as a cystadenoma or less likely peritoneal inclusion cyst. Serum tumor markers of CA-125 were elevated at 110 units/mL (reference range 0.5-35). CA-19-9, alpha fetoprotein, and carcinoembryogenic antigen were within normal limits. Gynecology-oncology was consulted for ovarian mass and recommended to follow up as outpatient for cystectomy with possible oophorectomy and salpingectomy, in addition to a diagnostic paracentesis for cytologic analysis. However, given our patient’s comorbidities, she opted not to pursue definitive diagnosis with surgery and instead sought symptomatic treatment. 

## Discussion

The combination of adnexal mass with pleural effusion and ascites in our case raised suspicion for Meigs’ syndrome. This syndrome is a rare disorder characterized by pleural effusion and ascites in patients with ovarian fibroma or fibroma-like tumors [6]. It was first described in 1887 by Demons, and later in 1937 by Meigs, who arrived at the same findings about association of pleural effusion, ascites, and benign ovarian fibroma. Hence, this syndrome is also known as Demons-Meigs' syndrome [7]. On the other hand, benign tumors of the ovary (other than fibromas) and ovarian malignancies, such as mature teratomas and struma ovarii, can also be associated with pleural effusions and ascites but they are categorized as pseudo-Meigs’ syndrome [3].

Krenke et al. reviewed pleural effusion characteristics in reported cases of Meigs’ syndrome. They found that the average amount of pleural fluid withdrawn was approximately three liters, and the majority of pleural effusions typically occurred on the right side. The majority of pleural effusions in patients with Meigs’ syndrome are exudative [8]. In our case, the pleural fluid analysis also revealed an exudative effusion with no malignant cells, consistent with the majority of Meigs’ syndrome cases. Review of reported cases of pseudo-Meigs syndrome shows that pleural fluid in these patients contains reactive mesothelial cells with no neoplastic cells [9].

Pleural effusion in Meigs’ syndrome is thought to be secondary to the passage of ascitic fluid to the pleural space through the diaphragm or diaphragmatic lymph nodes. Several hypotheses have been proposed for the underlying pathophysiology of ascites in these patients. Fluid leakage from edematous fibromas, tumor pressure on pelvic and abdominal lymphatics, and causing lymphatic blockage are among these theories [10]. Vascular endothelial growth factor (VEGF) that raises capillary permeability is also reported to be associated with formation of pleural and peritoneal fluid. Ishiko et al. reported a significant difference between the VEGF levels in the pleural and peritoneal fluid before and after tumor removal in patients with Meigs’ syndrome [11].

CA-125 antigen is a tumor marker associated with ovarian carcinoma. Nevertheless, elevated levels of CA-125 have also been reported in the literature for Meigs syndrome, although levels above 1,000 U/mL were rarely reported [2,12]. Lin et al. used immunohistochemical techniques to localize CA-125 expression, and they found that it is expressed by mesothelium rather than the fibroma [13]. Case reviews have shown that higher levels of CA-125 are associated with higher volume of ascites but size of tumor was not linearly correlated with CA-125 levels [14].

Following multidisciplinary evaluation, our patient decided to not pursue surgical intervention to remove the adnexal mass due to multiple comorbidities. Removal of the tumor will ultimately result in resolution of ascites, pleural effusion, and normalization of CA-125 in Meigs’ and pseudo-Meigs’ syndrome [1,15]. However, surgery may not be feasible options for all the patients. Patients with malignant pleural effusion and ascites or the ones who have significant comorbidities will not always choose to pursue adnexal mass resection. This is where symptom control and palliative medicine are sought out. Repeated large-volume paracentesis and/or thoracentesis have been palliative choices for these patients. Another option is abdominal indwelling peritoneal catheter placement, which is associated with the same complication rates but lowers separate patient encounters and increases patient satisfaction [16]. Repeated thoracentesis, indwelling pleural catheter (IPC), and pleurodesis are the available therapeutic modalities for management of symptomatic pleural effusion. Multiple studies have reported that IPC placement not only results in good symptomatic control but also can lead to spontaneous pleurodesis [17,18]. Therefore, it is important for providers to counsel Meigs’ syndrome patients who are not ideal candidates for surgery on all of the available palliative options for symptomatic relief.

## Conclusions

In this case report, we presented a patient who had pleural effusions, ascites, elevated CA-125, and an adnexal mass. These findings are consistent with the classical presentation of Meigs’ syndrome. However, definitive diagnosis was not made due to the patient’s preference of symptomatic treatment rather than definitive treatment with surgical intervention given her comorbidities. Therefore, it is important for providers to counsel Meigs’ syndrome patients who are not ideal candidates for surgery on all of the available palliative options for symptomatic relief.

## References

[1] Meigs JV (1954). Fibroma of the ovary with ascites and hydrothorax: Meigs' syndrome. Am J Obstet Gynecol.

[2] Timmerman D, Moerman P, Vergote I (1995). Meigs' syndrome with elevated serum CA 125 levels: two case reports and review of the literature. Gynecol Oncol.

[3] Peparini N, Chirletti P (2009). Ovarian malignancies with cytologically negative pleural and peritoneal effusions: demons' or Meigs' pseudo-syndromes?. Int J Surg Pathol.

[4] Quirk JT, Natarajan N (2005). Ovarian cancer incidence in the United States, 1992-1999. Gynecol Oncol.

[5] Kurman RJ, Carcangiu ML, Herrington CS, Young RH (2014). WHO Classification of Tumours of Female Reproductive Organs. https://publications.iarc.fr/Book-And-Report-Series/Who-Classification-Of-Tumours/WHO-Classification-Of-Tumours-Of-Female-Reproductive-Organs-2014.

[6] Shih JA, Garrett LA, Carbo AR (2019). Meigs' syndrome: a sheep in Wolf's clothing. Am J Med.

[7] Brun JL (2007). Demons syndrome revisited: a review of the literature. Gynecol Oncol.

[8] Krenke R, Maskey-Warzechowska M, Korczynski P, Zielinska-Krawczyk M, Klimiuk J, Chazan R, Light RW (2015). Pleural effusion in Meigs' syndrome-transudate or exudate?: systematic review of the literature. Medicine.

[9] Dunn JS Jr, Anderson CD, Method MW, Brost BC (1998). Hydropic degenerating leiomyoma presenting as pseudo-Meigs syndrome with elevated CA 125. Obstet Gynecol.

[10] Riker D, Goba D (2013). Ovarian mass, pleural effusion, and ascites: revisiting Meigs syndrome. J Bronchology Interv Pulmonol.

[11] Ishiko O, Yoshida H, Sumi T, Hirai K, Ogita S (2001). Vascular endothelial growth factor levels in pleural and peritoneal fluid in Meigs' syndrome. Eur J Obstet Gynecol Reprod Biol.

[12] Benjapibal M, Sangkarat S, Laiwejpithaya S, Viriyapak B, Chaopotong P, Jaishuen A (2009). Meigs' syndrome with elevated serum CA125: case report and review of the literature. Case Rep Oncol.

[13] Lin JY, Angel C, Sickel JZ (1992). Meigs syndrome with elevated serum CA 125. Obstet Gynecol.

[14] Liou JH, Su TC, Hsu JC (2011). Meigs' syndrome with elevated serum cancer antigen 125 levels in a case of ovarian sclerosing stromal tumor. Taiwan J Obstet Gynecol.

[15] Yadav S, Tomar R, Verma N, Khurana N, Triathi R (2017). Struma ovarii with pseudo-Meigs' syndrome and raised cancer antigen-125 levels masquerading as an ovarian carcinoma: case report and literature review. Sultan Qaboos Univ Med J.

[16] Rosenberg S, Courtney A, Nemcek AA Jr, Omary RA (2004). Comparison of percutaneous management techniques for recurrent malignant ascites. J Vasc Interv Radiol.

[17] Demmy TL, Gu L, Burkhalter JE (2012). Optimal management of malignant pleural effusions (results of CALGB 30102). J Natl Compr Canc Netw.

[18] Wahidi MM, Reddy C, Yarmus L (2017). Randomized trial of pleural fluid drainage frequency in patients with malignant pleural effusions. the ASAP trial. Am J Respir Crit Care Med.

